# No deterioration in health-related quality of life in patients with axial spondyloarthritis followed for 5 years in ordinary outpatient clinics in the biological treatment era

**DOI:** 10.1007/s11136-019-02308-4

**Published:** 2019-09-26

**Authors:** Gudrun Rohde, Kari Hansen Berg, Are Hugo Pripp, Anne Prøven, Glenn Haugeberg

**Affiliations:** 1 Faculty of Health and Sport Sciences, University of Agder, Norway and Department of Clinical Research, Sorlandet Hospital, Kristiansand, Postbox 422, 4604 Kristiansand, Norway; 2 Faculty of Health and Sport Sciences, University of Agder, Kristiansand, Norway; 3 Oslo Centre of Biostatistics and Epidemiology, Research Support Services, Oslo University Hospital, Oslo, Norway; 4 Faculty of Health Sciences, OsloMet – Oslo University Hospital, Oslo, Norway; 5 Department of Rheumatology, Martina Hansens Hospital, Baerum, Norway; 6 Division of Rheumatology, Department of Medicine, Sorlandet Hospital HF, Kristiansand, Norwegian University of Science and Technology, Trondheim, Norway

**Keywords:** Health-related quality of life, Axial spondyloarthritis, 5 years, Biological treatment era

## Abstract

**Background:**

Axial spondyloarthritis (ax-SpA) causes pain, fatigue, stiffness, loss of physical function and impaired health-related quality of life (HRQOL).

**Aims:**

The study aimed to explore the changes in HRQOL over 5 years in patients with ax-SpA and to identify baseline predictors associated with changes in HRQOL assessed using three HRQOL measures.

**Methods:**

Demographic, disease, medication and HRQOL data were collected at baseline and at 5-year follow-up. HRQOL was assessed using SF-6D, 15D and SF-36. Analyses involved McNemar, independent paired *t* tests and multiple regression.

**Results:**

In the 240 (women 31%, men 69%) ax-SpA patients assessed (mean age 46 years), measures reflecting disease activity decreased and co-morbidities increased, and more patients were treated with biologic drugs during follow-up. No deterioration in HRQOL was observed between baseline and 5-year follow-up; indeed, there was a significant increase in SF-6D and SF-36 PCS scores. Improvement in HRQOL measured by SF-6D was associated with younger age, higher education, low Bath Ankylosing Spondylitis (BAS) Activity Index (BASDAI), high BAS Patient Global Score and high C-reactive protein; improvement in SF-36 PCS was associated with younger age, higher education, low BASDAI and no use of biological treatment at baseline.

**Conclusion:**

Our ax-SpA outpatient clinic patients, with more patients treated with biologic drugs during the 5-year follow-up, did not deteriorate in HRQOL. In fact, the physical dimension in HRQOL improved over the years, as did measures reflecting disease activity. Our study adds evidence to the importance of suppressing inflammation to maintain and improve HRQOL in ax-SpA patients.

## Background

Axial spondyloarthritis (ax-SpA) is a chronic inflammatory disorder of the axial skeleton that causes pain, fatigue, stiffness, loss of physical function and impaired health-related quality of life (HRQOL) [1–4]. HRQOL is a subjective and multidimensional concept that can be defined as an individual’s experience of their general health state, including their physical, social and mental well-being [5]. HRQOL can be assessed using generic instruments (e.g. 15D and SF-6D) [6, 7] that are used in economic evaluation (cost–benefit analysis) and to calculate quality-adjusted life years (QALYs) [6, 7], and by other generic HRQOL measures such as SF-36 [8, 9].

Previous studies have shown that patients with ax-SpA report HRQOL scores that are similar to those reported for other inflammatory diseases [2, 10], but lower than those in healthy controls [1, 4, 11–13]. Women with the ax-SpA report lower HRQOL than men [14–16], and decreased HRQOL in patients with ax-SpA is associated with fatigue [17], increased disease activity, decreased daily activity and exercise [4, 18–20], pain, and adverse psychological factors such as body-image disturbance, anxiety and depression [21, 22]. In the new millennium, effective new treatments have become available for Ax-SpA [e.g. tumour necrosis factor (TNF) inhibitors] [23] and new treatment strategies have been recommended (the treat-to-target strategy) [23, 24].

Only a few studies have examined long-term changes in HRQOL in ax-SpA patients and explored predictors of changes in HRQOL [22]. Two previous studies found no long-term changes in HRQOL and no major deterioration in clinical outcomes [25, 26]. The aims of our study were to explore changes in HRQOL over 5 years in patients with ax-SpA and to identify baseline predictors associated with changes in HRQOL assessed using three different measures.

## Methods

### Patient recruitment

Patients with ax-SpA were recruited consecutively from two public outpatient rheumatology clinics, one located in the eastern part of Norway [Martina Hansens Hospital (MHH)] and the other in the south [Sorlandet Hospital (SSHF)]. We have previously reported cross-sectional data for this cohort at inclusion [4]. At inclusion, the patients were 18 years or older and all fulfilled the Assessment of Spondyloarthritis International Society (ASAS) criteria for ax-SpA [27]. As the patients were invited and included consecutively, only a small number of patients fulfilling the inclusion criteria choose not to take part in the study (4 at MHH and 4 at SSHF). Due to ethical reasons, we have no characteristics of the patients which choose not to take part.

### Data collection

The same data collection performed at baseline was also performed at the 5-year follow-up, including demographic, disease- and treatment-related variables, as described in detail previously [4]. Because of funding restrictions, the latest included patients at MHH were not invited for a follow-up examination. Among the 380 ax-SpA patients examined at baseline (MHH = 252 and SSHF = 128), 240 patients (63%) (MHH = 133 (53%) and SSHF = 107 (84%)) were re-examined after 5 years. The patients who did not participate in the 5-year follow-up data collection had significantly more co-morbidities at baseline than the patients who took part (0.93 (1.05) vs. 0.58 (0.81), *p* = 0.001). For other variables (apart from age), there were no significant differences between participants and non-participants at 5-year follow-up (data not shown).

Data were collected using interviews, questionnaires, laboratory tests and physical examinations. The demographic data collected by interviews included age, gender, education level (< 11 years, 11–13 years and > 13 years), work status (employed), physical exercise (exercise > 1 h per week/exercise < 1 h per week), body mass index (BMI) (kg/m^2^) and current smoker. Disease duration was defined as the time between the date at which patients fulfilled the ASAS criteria for ax-SpA and the date of their inclusion in the study, and human leucocyte antigen (HLA)-B27 status was recorded. Disease activity was assessed using the Bath Ankylosing Spondylitis Activity Index (BASDAI) (range 1–10), the Maastricht Ankylosing Spondylitis Enthesitis Score (MASES) (range 1–13) and C-reactive protein (CRP)(mg/dl) level. Physical function was assessed by the Bath Ankylosing Spondylitis Functional Index (BASFI) (range 0–10) and the Health Assessment Questionnaire (HAQ) (range 0–3) [28]. Data were also collected for the Bath Ankylosing Spondylitis Patient Global Score (BAS-G) (range 0–10) and morning stiffness. Current medications including the use of non-steroidal anti-inflammatory drugs **(**NSAIDs), synthetic disease-modifying antirheumatic drugs **(**DMARDs), biological DMARDs and prednisolone were also recorded. Finally, data on co-morbidities (yes/no) (heart diseases, pulmonary diseases; neurological, endocrine, haematological, gastro-intestinal, urogenital or other rheumatological diseases, mental disorders and cancers) were collected, and a summed score generated to reflect overall co-morbidity. This score has also been used in other studies [29–32].

To assess HRQOL, we used both the SF-36 and 15D questionnaires. SF-36 is a generic questionnaire that includes eight domains: general health, bodily pain, physical function, role limitations (physical), mental health, vitality, social function and role limitations (emotional). The eight domains can be combined into physical and mental sum scales that reflect physical [physical component summary (PCS)] and mental [mental component summary (MCS)] health. The SF-36 scales were scored according to published scoring procedures, and each scale was expressed using values from 0 to 100, with 100 representing excellent health [8, 9, 33, 34]. Regression analyses were performed to impute missing values in accordance with the instructions provided by the developer of the questionnaire [8, 9]. Patients with valid responses to SF-36 at both baseline and 5-year follow-up were included in the analyses for this study.

From the SF-36, we also generated the SF-6D utility score [7], based on 11 questions from the SF-36. The SF-6D utility scores range from 0.29 to 1.00, with 1.00 indicating ‘full health’. The Norwegian standard SF-36 version 1.00 was used to derive the SF-6D. Regression analyses were performed to impute missing values in accordance with the instructions published by the developer of the questionnaire [7, 8]. The psychometric properties of the SF-6D questionnaire have been validated in several languages [7].

The 15D questionnaire is a generic, multidimensional, standardised tool for evaluating HRQOL that can be used primarily as a single index measure but also as a profile utility measure. It assesses 15 dimensions describing the patient’s health status: mobility, vision, hearing, breathing, sleeping, eating, speech, elimination, usual activities, mental function, discomfort and symptoms, depression, distress, vitality and sexual activity [6]. Each dimension comprises one question with five response categories. A single utility index score is obtained by incorporating population-based preference weights to the dimensions [35, 36]. The utility scores fall between 0.0 (being dead) and 1.00 (no problems in any dimension). Regression analyses were performed to impute missing values in accordance with the guidelines published by the developer of the questionnaire [6]. The psychometric properties of this questionnaire have been validated thoroughly in other studies in several countries [6, 35, 36].

### Statistical analyses

Statistical analyses were performed using IBM SPSS Statistics v. 25 (IBM Corp., Armonk, NY, USA). Continuous variables are presented as mean and standard deviation (SD, in parentheses) and categorical variables as numbers and proportions (%). Paired sample *t* tests and McNemar tests were used to analyse differences between baseline and 5-year follow-up. To calculate the HRQOL change-scores, we subtracted baseline scores from 5-year follow-up scores (delta SF-6D, delta 15D, delta PCS and delta MCS). To further examine the differences in HRQOL between baseline and 5-year follow-up, we calculated the effect size by dividing the change-scores by their respective SD at baseline [37]. We applied Cohen’s standards for effect size values as small effect 0.2, medium effect 0.5 and large effect 0.8 [38].

Multivariable linear regression analysis, backward procedure (*p* = 0.20), was used to examine the demographic and disease-related variables at baseline that were associated with changes in HRQOL (delta SF-6D, 15D, SF-36 PCS and SF-36 MCS scores). The independent variables in the multiple analyses were chosen based on univariate associations with one of the delta HRQOL values (*p* < 0.10), clinical experience and factors found to be associated with HRQOL in previous studies [17, 22, 39]. These factors also resonate with the wider theory within the field [24, 40]. In the final multivariable model, we included the demographic variables (age, gender and education), disease activity (assessed by BASDAI), health status (assessed by HAQ, BASFI and BAS-G scores), damage (assessed by BASMI score) and current treatment, and adjusted for gender and HRQOL baseline scores. Using this model, the conditions for multivariable linear regression analysis were met. We also examined predictors of clinically significant improvement in HRQOL using cut-off values identified in previous studies (delta SF-6D > 0.041 [41], delta15D > 0.015 [42], delta SF-36 PCS and delta SF-36 MCS > 2.5 [43]) using logistic regression. The final tested variables are listed in Table 3, and the level of significance was set at *p* < 0.05.

### Ethical and legal aspects

The study was approved by the Regional Committee for Medical Research Ethics (REK # 4.2007.2152).

## Results

### Demographic and disease-related characteristics

Table 1 lists the variables recorded and compares baseline and 5-year follow-up data. At the 5-year follow-up, patients had significantly more co-morbidities [0.94 (1.1) vs. 0.57 (0.81), *p* < 0.001], lower CRP [5.7 mg/dl (10.7) vs. 10.0 mg/dl (13.7), *p* < 0.001], better scores on MASES [1.30 (2.29) vs. 3.37 (3.92), *p* < 0.001], better BAS-G [3.04 (2.62) vs. 3.70 (2.29), *p* = 0.002], better HAQ [0.45 (0.45) vs. 0.52 (0.46), *p* = 0.032], fewer were smokers [43 (19%) vs. 66 (28%), *p* < 0.001], fewer were employed [161 (69%) vs. 173 (74%), *p* = 0.028], more patients were using biological DMARDs [85 (35%) vs. 56 (23%), *p* = 0.001], and a tendency that less patients used NSAID [79 (35%) vs. 97 (40%), *p* = 0.066].

**Table 1:** Demographic data, disease markers, disease activity measures, damage, health status and co-morbidity in 240 patients with axial spondyloarthritis at baseline and at 5-year follow-up

Demographic	Baseline	5-year follow-up	*p* values
Age, years	46 (12)		
Married or cohabiting	185 (77%)	180 (77%)	0.728
Current smoker	66 (28%)	43 (19%)	< 0.001
Employed	173 (74%)	161 (69%)	0.028
Exercise > 1 h per week	210 (88%)	213 (90%)	0.618
BMI (kg/m^2^)	27.0 (4.5)	27.1 (5.5)	0.652
Education			0.374
< 11 years	30 (12%)	26 (11%)	
11–13 years	81 (34%)	78 (33%)	
> 13 years	128 (54%)	132 (56%)	
Co-morbidity			
Mean total score for co-morbidity (range 0–10)	0.57 (0.81)	0.94 (1.12)	< 0.001
Disease activity measures			
CRP (mg/dl)	9.97 (13.16)	6.69 (10.70)	< 0.001
68-tender joint count	0.52 (2.06)	0.25 (1.44)	0.100
66-swollen joint count	0.11 (0.68)	0.04 (0.23)	0.129
BASDAI	3.09 (2.10)	2.87 (2.20)	0.139
MASES enthesitis score	3.37 (3.92)	1.30 (2.29)	< 0.001
Damage			
BASMI	2.33 (1.94)	2.38 (2.06)	0.583
Health status			
BASFI	2.58 (2.16)	3.36 (2.20)	0.122
BAS-G	3.70 (2.29)	3.04 (2.63)	0.002
HAQ	0.52 (0.46)	0.45 (0.45)	0.032
Current treatment			
NSAID	97 (40%)	79 (35%)	0.066
Synthetic DMARDs	15 (6%)	14 (6%)	1.000
Biological DMARDs	56 (23%)	85 (35%)	0.001

McNemar tests were used to compare differences in categorical variables between baseline and 5-year follow-up, and paires-sample *t* tests for continuous variables

*BMI* body mass index, *BASDAI* Bath Ankylosing Spondylitis Activity Index (range 1–10), *MASES* the Maastricht Ankylosing Spondylitis Enthesitis Score (range 1–13), *CRP* C-reactive protein, *BASFI* Bath Ankylosing Spondylitis Functional Index (range 0–10), *HAQ* Health Assessment Questionnaires (range 0–4), *BAS*-*G* Bath Ankylosing Spondylitis Patients Global Score medication (range 0–10), *NSAID* non-steroidal anti-inflammatory drugs, *DMARDs* disease-modifying antirheumatic drugs. Categorical data are presented as number (%) and continuous variables as mean (SD)

### Changes in HRQOL between baseline and 5-year follow-up

At the 5-year follow-up, the patients reported significantly better HRQOL scores on SF-6D [0.710 (0.117) vs. 0.692 (0.119), *p* = 0.047] and SF-36 PCS [41.89 (9.8) vs. 39.84 (9.6), *p* = 0.001] compared with baseline. No significant changes were found in 15D or SF-36 MCS. For the eight SF-36 subdomains, the patients reported significantly better scores for bodily pain [54.3 (21.3) vs. 48.0 (20.5), *p* < 0.001] and physical function [75.7 (22.1) vs. 74.2 (19.8), *p* = 0.003] (Table 2).

**Table 2:** Health-related quality of life in patients with axial spondyloarthritis at baseline and at 5-year follow-up assessed by SF-6D, 15D and SF-36

	Baseline Mean (SD)	5-year follow-up	*p* value	Effect size
SF-6D	0.692 (0.119)	0.710 (0.117)	0.047	0.15
15D score	0.854 (0.090)	0.865 (0.138)	0.166	0.12
SF-36 PCS	39.8 (9.6)	41.9 (9.8)	0.001	0.21
SF-36 MCS	48.8 (9.5)	48.6 (9.6)	0.754	− 0.02
SF-36 eight domains				
Bodily pain	48.0 (20.5)	54.3 (21.3)	< 0.001	0.31
General health	55.1 (21.3)	57.5 (22.8)	0.058	0.11
Physical function	74.2 (19.8)	75.7 (22.1)	0.003	0.08
Physical role function	44.7 (41.8)	54.1 (43.5)	0.229	0.22
Mental health	78.0 (13.3)	78.5 (14.1)	0.592	0.04
Vitality	48.5 (19.9)	49.6 (22.0)	0.405	0.06
Social function	76.3 (21.9)	77.7 (21.9)	0.274	0.06
Emotional role function	74.5 (38.6)	75.4 (37.5)	0.749	0.02

Paired sample *t* tests were used to compare baseline and 5-year follow-up

The scores for SF-6D range from 0.29 to 1.00, with 1.00 indicating ‘full health’ and those for 15D range from 0.0 (being dead) to 1.00 (no problems on any dimension). The score for SF-36 ranges from 0 to 100 where 100 indicates a high HRQOL

*PCS* physical component summary, *MCS* mental component summary

### Baseline characteristics associated with 5-year changes in HRQOL

Improvement in HRQOL measured by SF-6D was associated with the baseline characteristics of younger age (*p* = 0.039), higher education (*p* = 0.008), low BASDAI (*p* = 0.009), high BAS-G (*p* = 0.007) and high CRP (*p* = 0.024). Improvement measured by 15D was associated with high CRP (*p* = 0.003), that assessed by SF-36 PCS with younger age (*p* = 0.036), higher education (*p* = 0.036), low BASDAI (*p* = 0.001) and no use of biological treatment at baseline (*p* = 0.027), and finally, that assessed by SF-36 MCS with higher education (*p* = 0.024) and low HAQ score (*p* = 0.029) (Table 3). Multivariable logistic regression analysis of clinically important improvement in HRQOL over the 5-year period identified the same patterns of associated variables (data not shown).

**Table 3:** Multivariable regression model reporting the associations between demographic and clinical variables and changes in HRQOL (delta SF-6D, delta 15D, delta SF-36 PCS and delta SF-36 MCS)

	Delta SF-6D	*p* value	Delta 15D	*p* value	Delta SF-36 PCS	*p* value	Delta SF-36 MCS	*p* value
	Final model stand B		Final model stand B		Final model stand B		Full model stand B	
Demographic factors								
Constant	0.435	< 0.001	0.136	0.459	1.33	0.910	27.66	< 0.001
Age	− 0.126	0.039			− 0.141	0.019		
Women								
Higher education (> 13 years)	0.160	0.008			0.126	0.036	0.134	0.024
Disease activity measure								
BASDAI	− 0.271	0.007			− 0.326	0.001		
Health status								
BASFI								
BAS-G	0.321	0.007			0.202	0.053		
HAQ	− 0.124	0.142	− 0.152	0.092			− 0.132	0.029
Damage								
BASMI			− 0.125	0.092				
CRP	0.136	0.024	0.216	0.004			0.105	0.077
Current treatment								
Current biological					− 0.015	0.027		
Current NSAID			− 0.091	0.198				
Current DMARDs								
Health-related quality of life—baseline								
F-6D	− 0.160	0.008						
15D			− 0.251	0.005				
SF-36 PCS					− 0.550	< 0.001		
SF-36 MCS							− 0.594	< 0.001
Adj *R*^2^	39%		5.4%		29.3%		33.7%	

The final model used a backward-step procedure to define the included variables

*BASDAI* Bath Ankylosing Spondylitis Activity Index (range 1–10), *CRP* C-reactive protein, *BASFI* Bath Ankylosing Spondylitis Functional Index (range 0–10), *HAQ* Health Assessment Questionnaires (range 0–4), *BAS*-*G* Bath Ankylosing Spondylitis Patients Global Score medication (range 0–10), *NSAID* non-steroidal anti-inflammatory drugs, *DMARDs* disease-modifying antirheumatic drugs. The scores for SF-6D range from 0.29 to 1.00, with 1.00 indicating ‘full health’ and for 15D range from 0.0 (being dead) to 1.00 (no problems on any dimension). The score for SF-36 ranges from 0 to 100 where 100 indicates a high HRQOL. *PCS* physical component summary, *MCS* mental component summary

## Discussion

Analysis of the 5-year changes in HRQOL in patients with ax-SpA identified significantly better scores for two of the four main HRQOL measures, SF-6D and SF-36 PCS, while despite increased co-morbidities there were no significant changes in SF-36 MCS and 15D. Baseline characteristics associated with higher 5-year changes in HRQOL across the measures were lower age, higher education, lower BASDAI and BAS-G, and for SF-36 PCS only, no use of biological treatments. The patients seem to have better disease control at the 5-year follow-up compared with baseline, indicated by better scores for measures reflecting disease activity such as CRP, MASES, BAS-G, HAQ and SF-36 bodily pain. One likely explanation for this is that more patients were treated with biologic DMARDs at the 5-year follow-up than at baseline. The increase in use of biologic DMARDs in our study is in line with the results from the Norwegian NOR-DMARD registry. In this registry in the period 2002–2011, the prescription rates for biologic DMARDs increased and disease activity improved in ax-SpA patient [40]. Further, in the 5-year period of our study the treat to target, recommendations also included ax-SpA [24].

To our knowledge, this is the first study since the introduction of the treat-to-target approach that assesses changes in HRQOL in patients with ax-SpA over a 5-year period using three different measures. In contrast to previous studies [22, 25, 26], we identified better long-term HRQOL within both SF-6D and SF-36 PCS and no changes in SF-36 MCS and 15D. Although the patients had increased co-morbidities at the 5-year follow-up, which we would have expected to influence HRQOL, this did not seem to be the case. The treat-to-target approach might also partly explain the better HRQOL scores [23, 24]. Better disease control implies better functionality, mobility and less structural damage, all of which have been shown previously to be associated with HRQOL [44].

The differences in changes over 5 years using different generic HRQOL measures might be attributable to the nature of the health items included and the way the questions were asked [6–9, 45, 46]. However, most generic instruments intended for HRQOL assessment, including SF-6D, 15D and SF-36 as used in the present study, contain at least some items that focus upon physical, emotional and social functioning [47]. The significant changes in SF-36 PCS and SF-6D might also be attributable to a better responsiveness compared with the other instruments [43]. However, the effect sizes of the changes were rather weak, and it could be argued that these differences are of limited clinical relevance [43].

Baseline self-reported outcome measures reflecting disease activity and burden, together with younger age and higher education, were significant characteristics associated with better long-term HRQOL. This indicates that a lower disease burden at baseline was important for long-term HRQOL. Stable disease and low disease burden have also been identified previously as associated with stable HRQOL over time [22, 25]. The new treatment-to-target approach implies that medications are adapted to an individual’s disease activity and hence influence their HRQOL [48]. This is underlined by better SF-36 bodily pain and physical function scores at the 5-year follow-up.

A higher education level was also associated with better long-term HRQOL. Some previous studies have identified associations between education level and HRQOL [22], but consistent with our findings Ward et al. [49] found a positive association between higher education level and high HRQOL in patients with ax-SpA. With respect to the effect of age, younger patients have had the disease for a shorter time and are thus likely to be less affected by years of disease and structural damage. Disease duration may also be difficult to calculate in patients with ax-SpA as patients may have had symptoms years before final diagnosis is established, e.g. in ankylosing spondylitis, a phenotype of ax-SpA, it may take 5–10 years before structural changes, a result of inflammation, become visible on X-rays [50]. With the introduction of magnetic resonance imaging, the inflammatory disease process in the sacroiliac joints in ax-SpA can be visualised years before structural changes become visible [51]. Further, there may also be a referral delay from general practitioners to rheumatologist that again may delay the diagnosis of ax-SpA.

Although most of the factors significantly associated with better HRQOL assessed by different instruments were the same, we also identified predictors associated with individual measures, such as the association of no use of biological DMARDs with SF-36 PCS only. This might also be attributable to the different items included in this part of the measure, its response options and also the structures of the scores [8, 9]. Other studies have also identified an association between biological treatment and HRQOL using SF-36 [48]. On the other hand, our findings could be explained by the limited need for such treatment in patients with low disease activity.

Despite an increased number of co-morbidities over the 5-year period, there seemed to be a trend among ax-SpA patients towards improving disease control, as reflected by lower disease activity, better self-reported functioning and less pain, and a healthier lifestyle as indicated by fewer smokers. The improvement in disease activity in our ax-SpA patients during follow-up may also be explained by a treat-to-target approach and health professional close collaboration with each patient, including encouragement to a healthier lifestyle and a more targeted medication [24].

### Methodological considerations

A strength of this study is its long-term design. Another strength is the use of three different HRQOL assessment tools. Further, the study included a relatively large number of patients followed over a 5-year period, and many variables (objective measures and generic and disease-specific patient-reported outcome measures) were included both at baseline and at the 5-year follow-up.

Weaknesses of the study are that data were collected at only two time points. Although we identified changes, the timing of these changes is unknown or unclear, and another two or three time points for data collection would have made the results clearer. This would have been especially beneficial when it comes to prior use of NSAID, synthetic or biological DMARDs in patients not on treatment at follow-up. The 140 patients who were not invited to 5-year follow-up or the panel attrition might have contributed different results. Previous studies show that panel attrition tend to be less healthy than patients taken part [52]. However, there were only minor baseline differences between those who attended for follow-up and those who were lost to follow-up. A given time for stopping the invitations to 5-year follow-up at one of the hospitals also increased the possibility for random panel attrition. Multivariable linear regression analysis, backward procedure, might produce an overfitted model. However, the explanatory variables chosen for the first step of the model include what can be considered as standard variables, like demographic variables (e.g. gender and age), and variables based on previous studies and theory within the field, along with strong associations in the univariate analyses, and we choose to include a relative high number of clinical variable (which also the high number of patients allows for). Furthermore, the results may be biased by unobserved heterogeneity which could be a threat to the validity of the findings. Adding the lagged dependent variable (HRQOL baseline score) as explanatory variable in panel data may cause biases [53]. However, the HRQOL baseline score is a clinical and theoretical important variable to predict the clinical development of patients. The time period from baseline to follow-up was long (5 years). We therefore choose to include it in the statistical analysis due to its clinical relevance and predictive ability.

It can be considered as both a strength and a limitation that self-reported outcome measures reflecting disease activity and burden were strongly associated with changes in HRQOL measured by generic questionnaires. While it seems that the disease-specific measures captured the actual disease burden, some of the items included in both the disease-specific and generic questionnaires are similar and therefore might be strongly correlated.

## Conclusion

In conclusion, no deterioration in HRQOL was found in this cohort of outpatient clinic ax-SpA patients treated with more biologic drugs during the 5 years of follow-up, despite their increased age and increased numbers of co-morbidities. In fact, the physical dimension in HRQOL improved over the years as did also measures reflecting disease activity. Our results are encouraging and indicate that clinical outcomes, including HRQOL, have improved over recent years for patients with ax-SpA treated in the biological treatment era. Our study also adds evidence to the importance of suppressing inflammation to maintain and improve HRQOL in ax-SpA patients.
